# MiRNA Dysregulation in Childhood Hematological Cancer

**DOI:** 10.3390/ijms19092688

**Published:** 2018-09-10

**Authors:** Jaqueline Carvalho de Oliveira, Gabriela Molinari Roberto, Mirella Baroni, Karina Bezerra Salomão, Julia Alejandra Pezuk, María Sol Brassesco

**Affiliations:** 1 Department Genetics, Federal University of Paraná, 80060-000 Paraná, Brazil; jaqbiomed@yahoo.com.br; 2 Department of Pediatrics, Ribeirão Preto School of Medicine, University of São Paulo, 14049-900 Ribeirão Preto, Brazil; gabi_cdl@hotmail.com (G.M.R.); mirella_baroni@yahoo.com.br (M.B.); karina_slm@hotmail.com (K.B.S.); 3 Programa de Pós-graduação em Farmácia, Anhanguera University of São Paulo, UNIAN/SP, 05145-200 São Paulo, Brazil; julia.pezuk@hotmail.com; 4 Departamento de Biologia, Faculty of Philosophy, Sciences and Letters at Ribeirão Preto, University of São Paulo, 14040-901 Ribeirão Preto, Brazil

**Keywords:** miRNA, cancer, children, leukemia, lymphoma, review

## Abstract

For decades, cancer biology focused largely on the protein-encoding genes that have clear roles in tumor development or progression: cell-cycle control, apoptotic evasion, genome instability, drug resistance, or signaling pathways that stimulate growth, angiogenesis, or metastasis. MicroRNAs (miRNAs), however, represent one of the more abundant classes of cell modulators in multicellular organisms and largely contribute to regulating gene expression. Many of the ~2500 miRNAs discovered to date in humans regulate vital biological processes, and their aberrant expression results in pathological and malignant outcomes. In this review, we highlight what has been learned about the roles of miRNAs in some of the most common human pediatric leukemias and lymphomas, along with their value as diagnostic/prognostic factors.

## 1. Introduction

Following the first microRNA (miRNA) discovery in 1993 [1], a constantly increasing number of miRNAs have been described and investigated. In 2002, the first miRNA dysregulation associated with human disease revealed deletion of miR-15 and miR-16 as a frequent event in chronic lymphocytic leukemia patients [2]. A year later, another couple of miRNAS, miR-143 and miR-145, were described as downregulated in colon adenocarcinoma [3].

In 2004, Calin and colleagues mapped 186 miRNAs and found that over 50% of miRNA genes are located at cancer-associated genomic regions or in fragile sites, and that those located in deleted regions are generally downregulated in cancer samples [4]. In the same year, let-7 was associated with shortened postoperative survival in lung cancer [5] and it was identified as a specific miRNA profile in B cell chronic lymphocytic leukemia [6].

Thenceforth, many miRNAs have been identified to have an essential role in human carcinogenesis and progression. Several reports have shown that miRNAs are central in cancer pathways by acting as “oncomiRs” or “tumor-suppressive miRNAs” and are often related to apoptosis, cell proliferation, angiogenesis, metastasis, and drug resistance [7,8]. Furthermore, miRNA regulation is dependent on the expression of its multiple mRNA targets, which are not always constitutively expressed; consequently, a unique miRNA may have different effects under diverse conditions [9].

The biological behavior of pediatric tumors is heterogeneous with several aspects distinguishing them from their adult counterparts, including their location, cellular environment, cell of origin, and genetic mutations. From treatment perspectives, they are also heterogeneous, ranging from generally curable low-grade tumors to highly aggressive forms that are usually fatal. Consequently, the identification of molecular markers that can effectively predict prognosis, and might contribute to the development of new therapeutic approaches, is still needed.

Like in adults, dysregulation of miRNAs is a hallmark in childhood cancer. Herein, we will compile current information about the role of miRNAs in the biology of leading hematological cancers in a pediatric setting.

## 2. Leukemias

Leukemia, a cancer of the bone marrow (BM) that hampers normal hematopoiesis, is the most common childhood malignancy, accounting for about 30% of all pediatric cancer. The two major subtypes seen in children are acute lymphoblastic leukemia (ALL), and acute myeloid leukemia (AML) [10,11], though a small fraction may present chronic myeloid leukemia (CML) and juvenile myelomonocytic leukemia (JMML) [12,13].

### 2.1. Acute Lymphoblastic Leukemia

ALL represents 80% of all leukemia types in children [10] and in recent decades clinicians have seen a significant improvement in event-free survival (EFS) rates, currently exceeding 80% in developed nations [14]. This advancement was notably facilitated by multiagent chemotherapy regimens and risk-adapted therapy, where the study of laboratory-based outcome variables consents the allocation of treatment [14,15].

**Figure 1:**
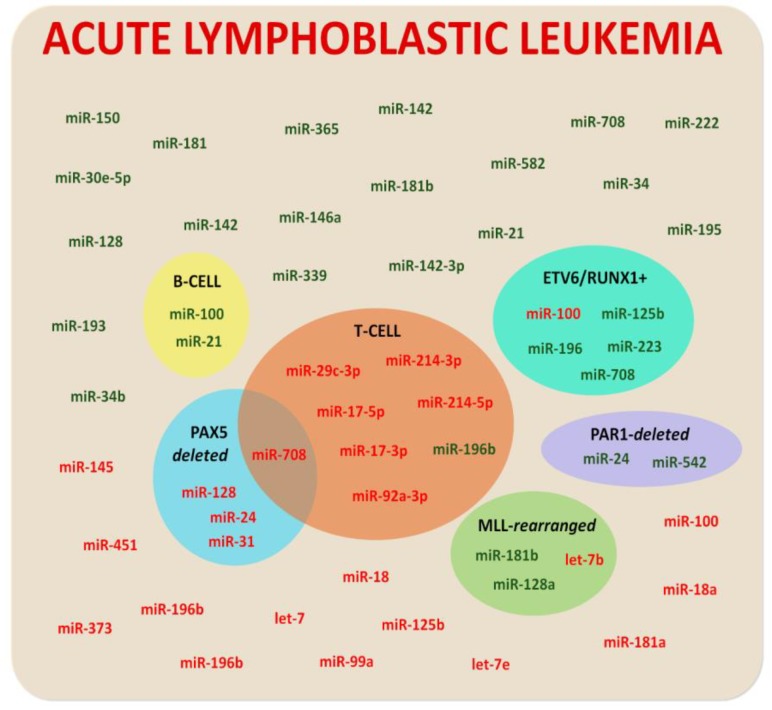
Dysregulated microRNAs (miRNAs) in childhood acute lymphoblastic leukemia. Hyperexpressed and hypoexpressed miRNAs in acute lymphoblastic leukemia within cellular (B-cell or T-cell) or molecular (*ETV6/RUNX1*+, *PAX5*-deleted, *MLL*-rearranged, or *PAR1*-deleted) subgroups are denoted in green and red, respectively.

In 2007, Mi and colleagues [16] showed that miRNA signatures could accurately discriminate ALL from AML. This study, although samples from adult and pediatric patients were analyzed indiscriminately, was the first suggestion of miRNAs’ involvement in childhood leukemia. Thereafter, many research groups also utilized miRNA-expression analyses and proved this strategy to be useful in the refinement of ALL classification schemes. Nowadays, disruption of miRNA expression and function in ALL is the most broadly studied and well-characterized among pediatric leukemias (Figure 1).

Differentially expressed miRNAs in childhood ALL (cALL) were firstly described in 2009 [17]. Examining 40 newly diagnosed pre-B ALL samples, miR-222, miR-339, and miR-142-3p were found overexpressed, along with the downregulation of miR-451 and miR-373 when compared to normal cells [17]. Additionally, a subsequent report [18] examined miRNA profiles in pediatric ALL samples in comparison to normal CD34^+^ cells, and gave evidence of the upregulation of miR-128a, miR-142, miR-150, miR-181, miR-30e-5p, miR-193, miR-34b, miR-365, miR-582, and miR-708, and the downregulation of miR-100, miR-125b, miR-99a, miR-196b, and miR-let-7e. Later on, several studies reported dysregulated miRNA expression in pediatric ALL samples compared to normal cells. For example, a number of publications described increased expression of miR-21, miR-34, miR-128, miR-142, miR-146a, miR-181b, miR-195, and miR-708 [19,20,21,22,23], and decreased levels of miR-18, miR-181a, miR-99a, miR-100, miR-145, let-7, and miR-196b in ALL cells [19,21,24,25].

The biological heterogeneity and distinct-lineage origins of ALL are well-established [26]. Such heterogeneity is also reflected with respect to miRNA expression profiles, for example, miR-18a is lower in childhood ALL when compared to the adult counterparts [24]. Among the dysregulation of miRNAs evinced in multiples studies, specific miRNA profiles have been described for specific ALL subtypes [18]. The same group [27] later identified unique miRNA expression patterns for each pediatric ALL subtype and measured the expression levels of 397 miRNAs in samples from 81 patients. The authors were able to differentiate many of the major subtypes of ALL, such as T-cell, *MLL*-rearranged, *ETV6*/*RUNX1*-positive, *E2A*/*PBX1*-positive, and hyperdiploid. However, conclusive evidence for discriminative miRNA expression was not found in *BCR*/*ABL* positive and “B-other groups”.

Moreover, the downregulation of miR-let-7b (~70-fold) in *MLL*-rearranged ALL; miR-100 in *ETV6*/*RUNX1*-positive; miR-17-3p, miR-17-5p, miR-29c-3p, miR-92a-3p, miR-214-3p, miR-214-5p, miR-708 in T-ALL; and miR-31, miR-24, miR-708, and miR-128 were associated with *PAX5*-deleted ALL [18,20,28,29]. Contrariwise, higher expression of miR-125b, miR-196, miR-223, and miR-708 were found in patients with *ETV6*/*RUNX1* translocation, and miR-24 and miR-542 were associated with PAR1 deletion, ALL [27,29,30] increased miR-181b and miR-128a in *MLL*-rearranged [20,27,31], higher miR-100 and miR-21 in B-ALL, and higher miR-196b in T-ALL [19,32,33,34]. Moreover, a difference on miRNA expression was found when infant and childhood T-ALL were compared [35].

**Figure 2:**
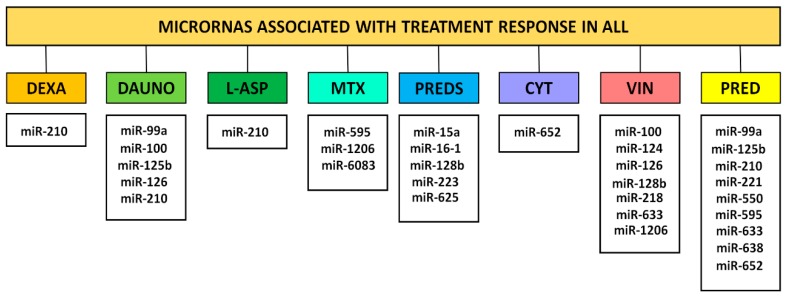
Dysregulated miRNAS that are associated with treatment response. Several miRNAs have been attributed an association with differential responses to dexamethasone (DEXA), daunorrubicin (DAUNO), l-asparaginase (l-ASP), methotrexate (MTX), prednisolone (PREDS), cytarabin (CYT), vincristine (VIN), or prednisone (PRED).

The clinical importance of miRNA profiling was also verified by the description of an association with treatment resistance and EFS. Specific miRNA profiles were described for several commonly used drugs (Figure 2). A study published in 2015 by Hamzeh et al. showed that miRNA-related dysregulated pathways were associated to resistance to asparaginase (L_ASP), daunorubicin (DAUNO), prednisolone (PREDS), and vincristine (VIN) [36]. Several miRNAs have been attributed an association with leukemia treatment resistance, such as miR-34 [37,38], miR-128b, and miR-223 [39]. Zhang et al. [23] described an miRNA signature (miR-18a, miR-532, miR-218, miR-625, miR-193a, miR-638, miR-550, and miR-633) that was able to predict prednisone (PRED) response in childhood ALL patients. Later, in a separate study involving cALL samples, it was shown that the expression profiles of the same group of miRNAs could similarly be used to predict early response to this glucocorticoid [40]. Furthermore, miR-124 was upregulated in prednisone and glucocorticoid resistance [41]. Alternatively, it was shown that prednisolone significantly increased miR-16-1 and miR-15a expression [42]. Additionally, it has been demonstrated that the restoration of miR-128b and miR-221 co-operatively sensitizes MLL/AF4(+) ALL cell lines to glucocorticoids [43], while exogenous expression of miR-335 in ALL cells renders cells to PREDS-mediated apoptosis [44].

Comparatively, in an attempt to elucidate miRNA signatures that indicate sensitivity to other chemotherapeutics used in ALL treatment, 397 miRNA were verified by Schotte and colleagues [27]. From those, 17 were related to resistance to one or more drugs. Among them, miR-99a, miR-100, miR-125b, and miR-126 were associated with VIN and DAUNO resistance, while miR-625 was associated with VIN and PREDS resistance. The expression of miR-125b, together with miR-99a and/or miR-100 overexpression, is also linked to vincristine resistance [45,46], while the overexpression of miR-652-3p increases sensitivity to vincristine and cytarabin (CYT) [47]. Recently, downregulation miR-326 was associated with multidrug resistance [48].

Furthermore, miR-3117-3p polymorphism has been associated with vincristine-induced neurotoxicity [49]. Moreover, miR-5189, miR-595, miR-6083, and some polymorphisms related to miRNA can also be linked to methotrexate (MTX) response, and miR-1206 can be used to predict MTX toxicity [50,51,52].

On the other hand, the association of distinct miRNA expression patterns in relation to risk stratification in childhood ALL has been scrutinized in the literature. A report by Zhang et al. [23] alluded to the association of miRNA expression with prognostic parameters such Central Nervous system (CNS) relapse, specific risk category, and disease recurrence. More than 20% of patients with CNS relapse showed a threefold increase of miR-7, miR-198, and miR-633, and a decrease of miR-126, miR-345, miR-222, and miR-551a at a one-year follow-up. Some of these findings were later confirmed in a report by Xu and colleagues [40].

The expression of some miRNAs can be used to monitor disease progression, such as with miR-128, miR-146a, miR-155, miR-181a, and miR-195 [21]. In addition, miR-210 has been proposed as a good prognostic factor and a useful predictor of drug sensitivity [53]. Moreover, a systematic investigation by Schotte et al. [27] verified a correlation with the probability of disease-free survival (DFS) and expression levels of 31 distinct miRNAs. Among those, 14 miRNAs were considered independent prognostic factors that allowed the distinction of a group of patients with favorable expression profiles and a five-year DFS of 89.4 ± 7% from those with less favorable miRNA profiles, with a five-year DFS rate of 60.8 ± 12%.

In parallel, Han et al. [54], using panel of matched samples (diagnosis/remission and diagnosis/relapse), described the altered expression of miR-223, miR-23a, let-7g, miR-181, miR-708, and miR-130b in relapsed samples, and miR-27a, miR-223, miR-23a, miR-181, and miR-128b in samples taken at remission. MiRNAs expression associated with shorter EFS or other clinical markers in cALL were also described [39,53,55,56,57,58]. An association of miR-708, miR-223, and miR-27a with relapse-free survival (RFS) was also demonstrated, as well as a prediction for relapse in patients with altered expression of miR-210 and for miR-143/miR-182 [59,60]. Moreover, the low expression of miR-151-5p and miR-451, high expression of miR-1290, or a combination of all three predicted inferior RFS [61].

### 2.2. Acute Myeloid Leukemia

**Figure 3:**
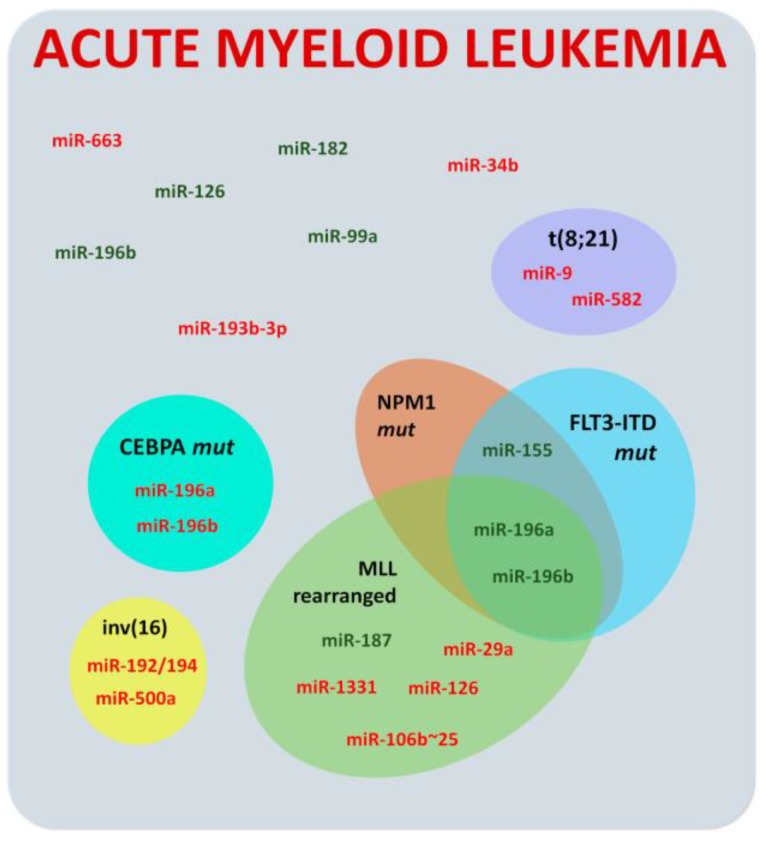
Dysregulated miRNAS in childhood acute myeloid leukemia. Hyperexpressed and hypoexpressed miRNAs in acute lymphoblastic myeloid and cellular/molecular subgroups are denoted in green and red, respectively.

Acute myeloid leukemia (AML) is the second most common type of pediatric leukemia, representing 17% of all hematological cancer in children [10] and five-year survival estimates of approximately 65% in developed countries [62]. MiRNA dysregulation has also been described in this pathology, though to a much lower extent when compared to ALL (Figure 3).

The first analysis of miRNA signatures in childhood AML (cAML) was also described by Mi and colleagues [16]. Nonetheless, adult and pediatric patients were analyzed indiscriminately and, among the 93 AML cases analyzed, only 17 were under 18 years old (nine patients under 12 years-old).

In 2009, samples from patients diagnosed with AML showed low levels of miR-34b, while in vitro exogenous expression of this miRNA caused cell-cycle abnormalities, reduced anchorage-independent growth, and altered CREB (cAMP response element-binding protein) target gene expression, suggesting suppressor potential [63]. Furthermore, in 2013, the same group demonstrated the hypermethylation of miR-34b promoter in AML [63].

Hypermethylation of the miR-663 promoter was also observed in another pediatric AML cohort and, consequently, a significantly lower expression of this miRNA was observed compared to normal bone-marrow control samples [64].

Several research groups have widely explored the study of individual miRNAs in AML. Emmrich et al. [65] observed that miR-582 and miR-9 are downregulated in t(8;21) AML; miR-500a and miR-192/194 are downregulated in AML with inv (16); and miR-181a, miR-1331, and miR-126 are downregulated while miR-187 is increased in *MLL*-rearranged AML. In 2017, Obulkasim et al. [66] published a signature prolife of 47 miRNAs to distinguished different AML cytogenetic subtypes. Moreover, miR-155 was proposed as a potential diagnostic biomarker for all AML, whereas miR-196b is specific for subgroups M4–M5 [67,68]. Another study stated that high miR-155 expression is also an adverse prognostic factor in pediatric NK-AML and is associated with worse EFS and overall survival (OS) [69].

In addition, miR-193b-3p was described as downregulated and proposed as an independent indicator for poor prognosis in pediatric AML, independent of patient age or genetics [70]. MiR-146b was described as an independent poor prognostic factor, while high expression of miR-181c and miR-4786 appeared to be favorable factors [71]. High expression of miR-196b in diagnostic marrow samples of pediatric AML was also associated with an unfavorable outcome [72].

Upregulation of miR-100 and miR-375 was also correlated with poor RFS OS [73], and downregulation of miR-29a was associated with advanced clinical features and poor prognosis of pediatric patients [74]. More, recently, an miRNA-based predictor of poststandard induction chemotherapy outcome in cAML was created to identify EFS in children with AML, and it offers the potential for improved patient stratification and management [75].

On the other hand, Danen-van Oorschot and colleagues [76] showed high levels of miR-196a and -b expression in pediatric patients carrying *MLL* fissions, *NPM1* mutations, or *FLT3*/*ITD*. In contrast, *CEBPA*-mutated cases presented low expression of miR-196a and -b. Alternatively, high expression of miR-155 was also observed in *FLT3*/*ITD* and *NPM1*-mutated cases, while downregulation of miR-29a was mostly detected in *MLL*-rearranged samples [76]. Moreover, the miR-106b~25 cluster has shown to be upregulated in relapse pediatric AML with MLL rearrangements [77].

Another comprehensive overview of miRNA expression showed that samples with core-binding factor AML and promyelocytic leukemia differed from each other and could be distinguished from *MLL*-rearranged AML subtypes by differentially expressed miRNAs that included miR-126, -146a, -181a/b, -100, and miR-125b [78].

MiR-99a was also found highly expressed in pediatric-onset AML, while significantly underexpressed during complete remission. Additionally, in vitro studies suggested a potential oncogenic role [79]. Moreover, forced expression of miR-9 reduced leukemic growth and induced monocytes differentiation of t(8;21) AML cell lines in vitro and in vivo, being characterized as a tumor-suppressor miRNA that acts in a strict cell context-dependent manner [65].

In parallel, many miRNAs have been related to AML by regulating cell proliferation, including downregulation of miR-122 as an aggressive progression marker [80], and miR-181a as a regulator of G1/S transition [81]. Others, like miR-126 and miR-182, were highly expressed in AML cell lines and inhibition of miR-126 significantly induced cell death through apoptosis [82,83]

### 2.3. Chronic Myeloid Leukemia

Chronic myeloid leukemia (CML) is a rare childhood hematological malignancy representing around 3% of all leukemias, with an annual incidence of one per million children and young people aged <15 years [84].

The characteristic reciprocal translocation t(9;22)(q34;q11) that leads to the formation of *BCR*/*ABL* chimeric oncoprotein is present in 90–95% of childhood CML [85]. In the era of therapy with specific tyrosine kinase inhibitors, the two-year survival among children with CML is 81–89%; nonetheless, age-group analysis evidenced that risk of death was three times higher for children younger than five years versus those aged 10–14 years [86].

Independent of age, many miRNAs have been described as active regulators of *ABL1* and *BCR*/*ABL*. For example, it was demonstrated that *ABL1* is a direct target of miR-203. This miRNA is silenced in CML and its restoration reduces *ABL1* and *BCR/ABL1* expression, decreasing cell growth. Additionally, one of the molecular mechanisms of imatinib is the demethylation of miR-203 in *BCR*/*ABL*-positive leukemia cells [87,88]. Nevertheless, the specific role of miRNAs in pediatric CML has been little explored. In 2013, miR-99a expression levels were evaluated in eight CML patients (including four samples before therapy and four samples with complete remission) and 12 pediatric controls. Although with a small number of patients, miR-99a expression was significantly increased in samples collected at diagnosis and decreased in samples after treatment [79].

A more recent study, aiming to evaluate profibrotic changes in childhood CML, analyzed 16 pediatric and 16 adult CML samples with and without fibrosis (each *n* = 8), as well as 18 non-neoplastic controls. Fiber accumulation in BM represents an adverse prognostic factor in adult CML, but, in children, this event is unknown. Nonetheless, among many gene-expression profiles investigated, two were miRNAs: miR-10 (previously associated with CML) and miR-146b (previously associated with fibrosis). MiR-10 was not associated with disease subtypes, fibrosis, or age. MiR-146b, on the other hand, showed lower expression levels in most pediatric samples when compared to adult counterparts, but no clear associations were found [89]. Other studies evaluating miRNA pathways specifically in childhood CML were not found.

### 2.4. Juvenile Myelomonocytic Leukemia

Juvenile myelomonocytic leukemia (JMML) is a rare myeloid progenitor disorder that occurs in young children with an annual incidence of as much as 1.2 per million children, accounting for less than 3% of all childhood hematologic malignancies [90].

Patients with JMML respond poorly to chemotherapy and have poor prognosis. The EFS is between 24–54% after hematopoietic stem-cell transplantation and is less than 10% without transplant [91]. Somatic defects in *RAS*, *NF1*, *PTPN11*, or *CBL* are detected in 85% of patients, evidencing RAS/MAPK-pathway (Rat Sarcoma virus /mitogen-activated protein kinase) activation as an important mechanism in JMML pathogenesis [92].

Aiming to evaluate miRNAs’ role in JMML, miR-let-7a-1/miR-let-7f-1 and the 3′UTR of *NRAS* or *KRAS* were sequenced in BM cells from 10 JMML patients. *RAS* is a known target of miR-let-7 but, in this report, there was no evidence of any mutations in let-7 or in let-7-binding sites that might lead to its upregulation in JMML [93]. On the other hand, reduced levels of most members of the miR-let-7 miRNA family were evidenced in a novel fetal-like subgroup of JMML patients with LIN28B protein overexpression [94].

Analyses of 20 JMML samples by Ripperger and coworkers through comparative genomic hybridization found two patients with an almost identical partial gain of chromosome 8, suggesting 8p11.21q11.21 as a critical region. This band includes 31 protein-coding genes and two noncoding RNAs among which miR-486 is a known regulator of phosphatase and tensin homolog (*PTEN*) and the transcription factor forkhead box O1 (*FOXO1*) [95].

In 2013, downregulation of miR-34b was described in JMML patients (*n* = 17) [63], but this association was not confirmed by Liu et al., analyzing a bigger JMML cohort (*n* = 47) [96]. Nonetheless, these authors described high expression levels of miR-183 (13.8 vs. 4.2, *p* < 0.001) with significant linear correlation with monocyte percentage and in samples with *PTPN11* mutations [96]. MiR-223 and miR-15a were also found upregulated in JMML BM harboring *PTPN11* mutations (11 from 19 analyzed patients), but not those without *PTPN11* defects [97].

More recently, distinctive miRNA signatures associated with the *PTPN11*, *KRAS*, and *NRAS* molecular subtypes of JMML were also described. From a panel of miRNAs, miR-630, miR-3195, miR-575, miR-4508, miR-224-5p, miR-320e, miR-494, miR-548ai, miR-222-3p, miR-23a-3p, and miR-338-3p were found upregulated, while miR-150-5p, let-7g-5p, miR-1260a, let-7a-5p, miR-4454, miR-148a-3p, miR-146b-5p, miR-342-3p, let-7f-5p, miR-26a-5p, let-7d-5p, miR-30b-5p, miR-29b-3p, and miR-29a-3p were described as downregulated. Of note, miR-150-5p was found to target *STAT5b* (Signal transducer and activator of transcription 5b), and its induced overexpression in mononuclear cells from JMML patients decreased proliferation rates [98].

## 3. Lymphomas

Lymphomas stricto sensu comprise any neoplasm of the lymphatic tissue. In the pediatric setting, lymphomas represent the third most common malignancy.

The World Health Organization (WHO) groups lymphomas by cell type and defining phenotypic, molecular, or cytogenetic characteristics [99]. Basically, there are two main categories of lymphoma, Hodgkin (HL) and non-Hodgkin lymphoma (NHL). HL most commonly affects adolescents and accounts for 4–7% of overall childhood cancer [100], while NHL is more frequently diagnosed in children younger than 15 years of age and represents 7–10% of pediatric malignancies [101,102]. NHL has a wide range of histological appearances and clinical features at presentation and, despite classification refinement, some groups remain heterogeneous. Nonetheless, Burkitt lymphoma (BL), diffuse large B-cell lymphoma (DLBCL), primary mediastinal large B-cell lymphoma (PMLBCL), anaplastic large cell lymphoma (ALCL), and lymphoblastic lymphoma (LL) comprise childhood NHLs [103].

**Figure 4:**
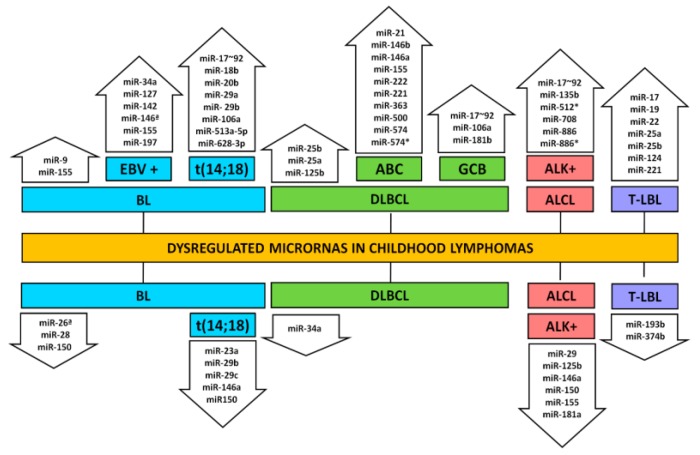
Dysregulated miRNAS in childhood lymphomas. Upside and downside arrows designate hyperexpressed and hypoexpressed miRNAs, respectively; Burkitt lymphoma (BL) (including Epstein–Bar virus and t(14;18) positive cases), diffuse large B-cell lymphoma (DLBCL) (including activated B-cell-like and germinal center B-cell-like subtypes), anaplastic large-cell lymphoma (ALCL) (including the ALK^+^ subtype), and lymphoblastic T-cell lymphoma (T-LBL).

Over the last decade, the pattern of miRNA expression of different types of pediatric lymphomas has been extensively studied. A substantial number of miRNAs have been described as dysregulated and contributing to better classifying this type of tumor, though, for many, their roles in tumor development are still unclear (Figure 4). Moreover, the lack of information about miRNAs in some forms, such as PMLBCL and classical HL, is evident.

### 3.1. Burkitt Lymphoma

BL represents about 30% of all pediatric NHL and is considered a highly aggressive tumor [102]. The study of miRNA in BL is usually focused on the establishment of miRNA profiles and the understanding of tumor transformation and progression. On this regard, miRNA expression could discriminate BL from other lymphomas, pediatric from adult samples, and Epstein–Barr virus (EBV) BL-positive from negative cases [104,105,106,107,108].

In 2004, miR-155 was the first described as deregulated in lymphoma. This miRNA is encoded by the human *BIC* gene and it was found overexpressed in pediatric BL. Metzler et al. [102] suggested that this RNA could be acting in co-operation with c-Myc on B-cell transformation. MiR-155 expression induces polyclonal expansion in B-cells, favoring the occurrence of secondary mutations and leading to full transformation [109]. This miRNA has as a direct target *SHIP1* (Src homology-2 domain-containing inositol 5-phosphatase 1), whose suppression in hematopoietic cells leads to mieloproliferative diseases [110]. In addition, miR-155 showed low expression in pediatric BL, inversely associated with the downregulation of the nuclear interactor of ARF (ADP-ribosylation factor) and Mdm2 (murine double minute 2) (NIAM, the protein-coding transcript splice variant of *TBRG1* locus), a protein with a tumor-suppressor function [111].

It was demonstrated that miR-155 was expressed only in BL EBV-positive cases, which account for 70% of all pediatric BL [112,113]. Secreted vesicles (exosomes) from EBV-positive Raji cells could deliver miR-155 to other recipient cell lines, such as retinal-pigment epithelial cells (ARPE-19), and miR-155 increased transcriptional and translational levels of VEGF-A in ARPE-19 cells [114].

Other studies have also demonstrated a close association between EBV infection and miRNA dysregulation in BL. High levels of miR-155 and miR-146a were found in response to the viral latent membrane protein-1 (LMP1) through NF-κB (nuclear factor kappa B) modulation, although the precise mechanism is still unclear [115,116]. LMP1 seems to stabilize BIC mRNA via p38/MAPK, and the LMP1–BIC axis contributes to EBV-induced lymphomagenesis [117]. Additionally, LMP1 induces miR-34a expression, leading to EBV-transformed cell growth [118]. However, a tumor-suppressor effect of LMP1 has also been described through the upregulation of miR-29b, which represses *TCL1* (T-cell leukemia/lymphoma protein 1) and leads to tumor cell-proliferation reduction [119].

Epstein–Barr nuclear antigen 1 (EBNA1) also has a role on BL development by miR-127 induction. This miRNA impairs B-cell differentiation by decreasing *BLIMP-1* (PR domain zinc finger protein 1) and *XBP-1* (X-box binding protein 1) expression, which leads to BCL-6 overexpression and IRF-4 (interferon regulatory factor 4) downregulation [120]. The presence of the EBV virus determines a profile of miRNAs in pediatric and adults BLs, therefore 28 miRNAs were differentially expressed in positive EBV cases, including EBV-encoded and host miRNAs [107].

During EBV infection, the virus-control miRNAs expression of host cells and expressed two clusters of miRNAs. A member of the miR-BART cluster, EBV-BART-6-3p target interleukin-6 receptor (IL-6R) and impair the immune system [121,122]. Synergistically with EBV-BASRT-6-3p, host cellular miR-142 and miR-197 targeted and reduced expression of IL-6R in Ramos BL cell lines [123,124]. Therefore, miRNA detection could be a specific and sensitive tool to recognize EBV vestiges once EBV-negative samples classified by immunohistochemistry demonstrated the presence of EBV-miRNAs, suggesting that EBV might contribute to lymphomagenesis [125].

Among the dysregulated miRNAs in BL, there are also those regulated by the NF-κB pathway and those regulated by c-Myc, transcription factors that regulate cell proliferation, growth, and apoptosis. Among these, miR-23a, miR-26a, miR-29b, miR-30d, miR-146a, miR-146b-5p, miR-155, and miR-221 were found statistically significantly downregulated in BL compared to other lymphomas [126]. In Raji cells, miR-520a was associated with the regulation of the AKT1 (v-akt murine thymoma viral oncogene homolog 1) and NF-κB signaling pathways, and mimics of this miRNA inhibited growth and proliferation, and promoted apoptosis [127].

Alterations on c-Myc expression or function of are one of the most frequent abnormalities in human malignancy; in BL, the recurrent t(14;18) chromosomal translocation juxtaposes this oncogene to the regulatory elements of the immunoglobulin resulting in the constitutive expression of c-Myc [128]. This activation promotes the expression of cluster miR-17–92 [129], which has a causative role in lymphomagenesis, regulating proapoptotic proteins and cell-cycle regulators [130]. In the p53-mutated BL cell line Raji, c-Myc is an alternative target of Inauhzin (INZ) via miRNA pathways, including miR-24 and miR-34a. INZ is a small molecule that activates p53 and inhibits tumor growth [131]. In fact, De Falco et al. (2015) showed four miRNAs (miR-29a, miR-29b, miR-513a-5p, and miR-628-3p) differentially expressing between MYC translocation-positive and negative BL. These miRNAs targets are involved in gene expression, proliferation, and DNA modification. In MYC translocation-negative, overexpression of DNA methyltransferase (DNMT) was associated with hypoexpression of the miR-29 family [132]. Association between high expression of DNMT1 and decrease in the miR-29 family was observed in a pediatric cohort (*n* = 71), suggesting a methylation control of has-miR-29 [133].

Analysis of known Myc-targeted miRNAs demonstrated significant association between BL with Myc translocation in a cohort composed by 61% of pediatric BL [104]. Those miRNAs included an upregulated cluster (miR-17-92 and its paralogs miR-18b, miR20b, miR-106a), and a set of downregulated miRNAs (miR-23a, miR-29c, miR-29b, miR150, miR146a). Upregulated miRNAs in BL were expressed at significantly lower levels in normal B cells, T cells and stromal cells, but were noted in BL cell lines (Daudi and Raji). BL cases with high expression of miR-17-92 cluster members showed significantly repression of these target genes in BL [104]. The investigation of the miR-17–92 cluster (miR-17, miR-19a, miR-19b, miR-20, and miR-92 a) expression in pediatric BL showed correlation between the upregulation of miR-17 and miR-20a with a lack of proapoptotic BIM (Bcl-2-like protein 11) expression. MiR-17 was a predictor of shortened OS, and inhibition of this miRNA in Daudi cells induced BIM expression [133].

MiR-26a and miR-28, on the other hand, were found underexpressed in BL. Their ectopic expression reduces proliferation, impairs cell-cycle progression, and increases apoptosis by targeting different proteins [134,135]. Another important c-Myc target, miR-150, which targets *MYB* and *survivin*, was found downregulated in BL. Overexpression of this miRNA in BL cells reduced proliferation rates and increased apoptosis [136,137].

Moreover, miR-181b, which is located in the intron of the *FAMLF* (familial acute myelogenous leukemia related factor) gene, showed an inverse correlation with FAMLF expression, and an interaction with its 5′UTR. Downregulation of *FAMLF* by miR-181b inhibited cell viability and arrested cell cycle in Raji BL cells [138,139].

Differential miRNA profiles have also been described in endemic Burkitt Lymphoma (eBL), an aggressive germinal center CG cell cancer that represents a subdivision of BL with high incidence in pediatric patients in equatorial Africa. eBL is associated with EBV and *Plasmodium falciparum* malaria coinfection, and shows c-Myc overexpression [140]. An integrative analysis compared normal germinal center (GC) B cells with eBL and evidenced 49 miRNAs with differential gene expression. Of these, 27 miRNAs were downregulated (including let-7 family members) and 22 upregulated (among them miR-17–92 cluster) in eBL samples. Enrichment of pathways showed the interaction of these miRNAs with marked tumor suppression (*PTEN*, *AXIN1*, *ATM*, *NLK*), and important proto-oncogenes and tumor-promoting genes as *MYC* [141].

A comparison between eBL jaw- and abdominal-tumor biopsies showed no discernible clustering based on tumor-site designation. MiR-10a-5p was the only miRNA with differential expression, and it was lower in jaw eBL compared to abdominal, and it presented reduced expression in nonsurvivor patients. MiR-10a-5p could target 473 genes, and enrichment of pathways showed its importance in cancer, focal adhesion, EPV infection, and apoptosis pathways [142]. Therefore, in pediatric BL, EBV infection and c-Myc translocation promote lymphomagenesis through the deregulation of several miRNAs.

### 3.2. Diffuse Large B-Cell Lymphoma

DLBCL represents 10% to 20% of childhood lymphomas and it is more frequently found in children older than 10 years of age [128]. This tumor is divided in two distinct subtypes according to the cell of origin: the activated B cell-like (ABC) and the germinal center B cell-like (GCB) [143], though most pediatric DLBCL patients are diagnosed with the GCB form [144].

The clinical presentation of pediatric DLBCL and BL are very similar. Currently, they are recognized as two different entities; however, in the pediatric group, there is a significant overlap of features resulting in a group of unclassifiable lymphomas. Nonetheless, through microarray technology, different research groups were able to define a collection of distinct miRNAs that constitutes a DLBCL signature [106,145]. This analysis also enables us to differentiate ABC from GCB-subtypes.

Ten miRNAs (miR-146b, miR-146a, miR-21, miR-155, miR-500, miR-222, miR-221, miR-363, miR-574, and miR-574*) were found to be more upregulated in ABC than in the GCB lymphoma type, suggesting that the high levels of these miRNAs are not due to tumor malignancy but associated with the cell of origin [146,147]. MiR-155 was one of the first miRNAs found upregulated in ABC-DLBCL [147,148]. Its aberrant expression seems to be a consequence of an autocrine stimulation by TNFα (tumor necrosis factor alfa) rather than chromosomal translocations like in BL tumors [149]. Initially, no correlation with prognosis was found when all DLBCL tumors were considered [145,150]; however, when only the ABC group was examined, high miR-155 expression was associated with better survival rates. In this case, the five-year survival probability changed from 15% for patients with low miR-155 to 53% for patients with high miR-155. Moreover, higher expression of miR-222 was also associated with inferior overall and progression-free survival [150].

Other studies later indicated miR-155 and miR-146a as potential diagnostic and prognostic indicators in DLBCL. Patients with low expression of these miRNAs were associated with high complete remission, high overall response rate, and better five-year OS when patients were treated with the R-CHOP protocol (rituximab, cyclophosphamide, doxorubicin, VIN, and PREDS) [151]. Furthermore, in DLBCL patients, higher expression of miR-28, miR-214, miR-339*, and miR-5586 was associated with better outcome, while upregulation of miR-324 was associated with poor prognosis [152].

Although ABC-DLBCL miRNA signatures have been better studied, the expression of few miRNAs was also associated with GCB-DLBCL, such as the amplification of the 17–92 cluster [153] and high levels of miR-106a and miR-181b [154].

Additionally, the role of some of these miRNAs in DLBCL development has been elucidated over the last few years. MiR-125a and miR-25b, for example, are overexpressed in DLBCL and target *TNFαIP3*, an NF-κB negative regulator. They participate in a positive self-regulatory loop where miR-125 is also regulated by NF-κB, what is probably an important mechanism to keep the constitutive activation of the NF-κB pathway in DLBCL pathogenesis [155]. In addition, miR-34a repression was described to cause high-grade transformation of B-cell lymphoma by altering *FOXP1* (Forkhead Box P1) expression [156]. Interestingly, mice treated with miR-34a mimics results in a 95% reduction in DLBCL tumor growth due to its strong proapoptotic properties, suggesting an alternative therapeutic strategy [157].

### 3.3. Primary Mediastinal Large B-Cell Lymphoma

PMLBCL was first described in the 1980s and is considered a distinct clinicopathologic entity of DLBCL [158]. PMLBCL is characterized by a rapidly growing mediastinal mass that arises from mature thymic medulla B-cells, frequently accompanied by local invasiveness and occasionally with distant metastasis. Uncommon, but not rare, this clinicopathological entity occurs more often in young adult females [159] and constitutes 2–3% of all N and 6–10% of all diffuse large-cell lymphomas [160,161].

Recently, a large population-based study was able to estimate the incidence of PMLBCL. Based on slightly more than 400 patients in the United States, the annual incidence rate was estimated at 0.4 per million. Females had significantly higher incidence than males (ratio 3:1) and the peak of occurrence was recognized at 30–39 years. The five-year survival rate of 85% and prognosis were also reduced with advancing age [162].

In patients aged <18 years (22/451 cases), PMLBCL incidence was 4.9% [162]. Other reports, in which young patients showed inferior outcomes, compared adult counterparts or children with other B-NHL histological subtypes, with EFS rates ranging between 70% and 80% [163,164,165,166,167].

In the literature, there are few studies on miRNA expression profiles in PMLBCL. The first report was given by Kluiver et al. [168], who showed positivity for *BIC* and miR-155 in one cell line and eight PMLBCL samples derived from a tissue bank. Later, Iqbal et al. [104] described an miRNA signature that allowed the distinction between PMLBCL from other DLBCL, including upregulation of miR-193b and miR-365, and underexpression of miR-629, miR-423-5p, and miR-15a. Higher levels of miR-92a were also described as a classifier of PMBLBCL [169]. Moreover, a recent study by Malpeli et al. [170] showed that the polycistron miR-17–92 cluster, miR-29 family, miR-150, and miR-497 had the highest power of discrimination between B-cell NHL types, though only eight PMLBCL samples were included. Nonetheless, none of these studies gave any specific details about pediatric samples, and the mean age of patients was always reported above 27 years old.

### 3.4. Anaplastic Large-Cell Lymphoma

Anaplastic large-cell lymphoma (ALCL) is an intermediate grade NHL and accounts for approximately 10% of pediatric NHL [171]. Most pediatric ALCL present the chromosomal translocation t(2;5) (p23;q35). In 80% of cases, that translocation results in the expression of a fusion gene called NPM–ALK that encodes a potent oncogenic tyrosine kinase [172].

Several miRNAs have been described as promoting this neoplasia and they seem to express and act differently in ALK^+^ and ALK^−^ tumors and cell lines [173,174]. The suggested signature for ALK^+^ cells includes seven miRNA, five of them being upregulated (miR-512*, miR-886, miR-886*, miR-708, and miR-135b) and two downregulated (miR-146a and miR-155). High expression of miR-886 and miR-886* seems to be related with higher AKT expression, since treatment with AKT inhibitors leads to a reduction on these miRNAs levels. It has been shown that miR-886 might act deregulating apoptosis by targeting the proapoptotic gene *BAX* [173]. Furthermore, miR-16 is downregulated in AKT^+^, resulting in VEGF expression, tumor growth, and angiogenesis [175].

Besides the above-mentioned miRNAs, the 17–92 cluster has also been found overexpressed in AKT^+^ ALCL [176]. These miRNAs are transcriptionally regulated by STAT3, a major substrate for ALK, and promote survival and growth of this tumor. Among the known targets of this cluster, BIM and TGFβRII have been described. An autoregulatory loop between STAT3 and miR-17–92 was also characterized, suggesting an involvement of this cluster in the pathogenesis of this tumor [177].

Conversely, miR-155 showed low expression in ALCL ALK^+^ tumors and cell lines, and its inhibition is mediated by methylation. SR278 transfection (pediatric ALCL ALK-positive cells) with pre-miR-155 reduced expression levels of miR-155 targets (C/EBPβ, SOCS1) by binding sites in their 3′-UTR. The action of miR-155 in the immune system was demonstrated through reducing IL-8 and IL-22 transcript levels [178]. C/EBPβ downregulation evidenced the role of this transcription factor in miRNA regulation, mainly miR-181a*, miR-181, and miR-203. MiR-181a showed low expression in ALK^+^ ALCL cases; this miRNA coordinates T-cell differentiation and modulates TCR antigen expression, being involved in innate and adaptive immune response [174].

MiR-29a was found remarkably reduced in ALK^+^ when compared to ALK-ALCL, where it regulates MCL-1, contributing with apoptosis blockage [179]. Moreover, ALK knockdown results in increased miR-96 levels, while miR-96 overexpression leads to a reduction in ALK protein levels and decreases cell viability and growth, reinforcing the hypothesis that ALK sustains its own expression by exerting a reciprocal negative feedback loop that hinders the expression of miRNAs [180]. ALK-positive cells showed low levels of miR-146a, miR-29c, miR-29b, miR-29a, miR-22, miR-101, miR-150, and miR-125b, while miR-20b was upregulated [176].

The translocation and activity of NPM-ALK are responsible for miR-150 and miR-125b silencing in cell lines, mediated by DNMT1-dependent activity [181,182]. Inhibition of DNMT1 binding to the MIR125B1 promoter decreased BAK1 expression, an miR-125b target. Mir-125b repression and increase of BAK1 is correlated with early relapse in human ALK^+^ ALCL biopsies [182]. Conversely, miR-101 is found downregulated in both types of ALCL, but, because it targets the mammalian/mechanistic target of the rapamycin (mTOR) pathway, its forced expression only affects ALK^+^ cell growth [176].

### 3.5. Lymphoblastic Lymphoma

LL is a rare neoplasm of immature cells committed to the B (B-LBL)- or T-cell lineage (T-LBL) that accounts for approximately 2% of all lymphomas. The annual incidence in children (<15 years) is 3.6 per 100,000, which is then reduced to 0.8 in people older than 25 years old [183]. Studies about the role of miRNAs on this form of lymphoma are scarce and are summarized as follows.

#### 3.5.1. B-Cell Lymphoblastic Lymphoma

B-LBL typically affects children younger than six years, but is also encountered in older children and in adult populations [184]. B-LBL tumor cells are virtually always positive for B-cell markers CD19, CD79a, and CD22, and may be associated with the presence of leukemia rearrangements such as those involving *ETV6*, *MLL,* or *ABL1* [183]. Thus, even though lymph nodes and extranodal sites, such as skin, bone, and soft tissue, are frequently involved, this rare NHL is considered a lymphomatous variant of ALL and is often treated with leukemia-like regimens [183].

As a result, over the last years, high priority has been given to the identification of biological/prognostic features of T-LBL to allow either risk stratification or treatment planning.

#### 3.5.2. T-Cell Lymphoblastic Lymphoma

T-LBL represents 30% of pediatric NHL [185]. The downregulation of miR-193b in T-LBL was first associated with the activation of the GLI/hedgehog pathway promoting cell survival and proliferation by enhancing SMO (smoothened) expression [186]. Later, this miRNA with miR-196b were found involved in the regulation of the PDGF (platelet-derived growth factor) signaling pathway. In addition, miR-221 was specifically found upregulated in T-LBL directly targeting CDKN1B, a cell-cycle regulator [187]. MiR-22, miR-125a, and miR-125b were also identified as upregulated in T-LBL, and seem to have a role on the maintenance of hematopoietic cells contributing to their proliferation and self-renewal abilities [187].

In a cohort with 52% of T-LBL pediatric samples, upregulation of miR-17 and miR-19 and positive MYC protein was associated with unfavorable prognosis. MYC is known to regulate the miR-17–92 cluster. Cox proportional hazard models showed that miR-17, miR-19, and MYC overexpression were independent poor prognostic factors [188]. MiR-241 is upregulated in T-LBL tissue and a direct target of a long noncoding RNA *MEG3* (maternally expressed 3). Overexpression of MEG3 inhibits tumor growth in vitro and in vivo [189].

Downregulation of miR-374b was associated with worse overall survival and increased risk in T-LBL samples. MiR-374b inhibited proliferation and promoted apoptosis in a pediatric T-LBL cell line (SUP-T1) by repressing AKT1 and Wnt-16 [190]. Moreover, upregulation of miR-221-3p and miR-222-3p, and downregulation of miR-203a and miR-205-5p, miR-200a-3p, and miR-375 have shown to play important roles in T-LBLs by dysregulating in the CDKN1C/E2F1/TP53 axis [191].

### 3.6. Hodgkin’s Lymphoma

Hodgkin lymphoma (HL) is characterized by multinucleated giant cells (Hodgkin/Reed-Sternberg cells, H/RS) or large mononuclear cell variants (lymphocytic and histiocytic cells) (representing 1% of the tumor) in a background of inflammatory cells that include lymphocytes, histiocytes, neutrophils, eosinophils, plasma cells, and fibroblasts [192]. The annual incidence of HL is 2–3 cases per 100,000 in Europe and the USA, with a bimodal peak, with young adults aged 15–34 being the most affected, followed by those aged 60 and older [193]. HL accounts for 5% to 6% of all childhood cancer and is one of the most curable forms [194]. The five-year EFS in childhood and adolescence exceeds 90% for patients with early-stage and 70% to 80% for those with advanced-stage disease [195].

Over the last decade, efforts have been made in order to identify miRNAs as biomarkers for the refinement of diagnosis and therapy of HL; even so, information is still limited. Some miRNAs have been described as dysregulated in adult samples (mean age 29 years old) by different groups [196,197,198]. MiR-25, miR-30a/d, miR-26b, miR-182, miR-186, miR-140*, and miR-125a [199], or miR-34a-5p, miR-146a-5p, miR-93-5p, miR-20a-5p, miR-339-3p, miR-324-3p, miR-372, miR-127-3p, miR-155-5p, miR-320a, and miR-370 [200], for instance, have been described as upregulated in tumor samples. Concomitantly, miR-23a, miR-122, miR-93, and miR-144 [199], miR-582-3p, miR-525-3p, miR-448, miR-512-3p, miR-642a-5p, miR-876-5p, miR-532-3p, miR-654-5p, miR-128, miR-145-5p, miR-15b-5p, miR-328, and miR-660-5p were designated as downregulated [200]. Other studies that are based on a limited number of samples have no information about age [201], used data miming [202], or are centered on different cell lines that are all of adult origin [203,204,205,206]. Thus, so far, there is no information about dysregulated miRNAs in the pediatric setting.

## 4. MiRNAs in Clinics

### 4.1. Circulating MiRNAs as Biomarkers

The pursuit for noninvasive tools for the diagnosis and management of cancer has long encouraged the interest of researchers into the field of circulating nucleic acids. Compelling evidence has shown that genetic and epigenetic cancer markers are also measureable in the plasma and serum of cancer patients and may be useful as a tool for early detection, diagnosis, and follow-up [207,208].

Recently, extracellular circulating miRNAs were detected in secreted membrane vesicles (exosomes), blood serum, and other body fluids. This discovery suggests that miRNAs play a role in intracellular communication in both a paracrine and endocrine manner [209]. Dysregulated expression of miRNAs is implicated in tumorigenesis; therefore, functional characterization of these miRNAs in cancer has received more attention in identifying promising diagnostic and/or prognostic biomarkers. On this regard, MiRNAs are ideal candidates due to their unique expression patterns associated with disease-stage stability and their stability in plasma, easy detection, and recovery [210].

After the first description of circulating miRNAs in lymphoma patients, a significant increase in the number of studies appeared [207], but the amount of research of pediatric cases is smaller than their adult counterparts.

In ALL, a few circulating miRNAs were recently described, and among them miR-146a. Significant higher median levels of miR-100, miR-196a, and miR-146a were reported in blood samples of affected children compared to controls, but the diagnostic efficacy for miR-146a analysis presented superior sensitivity and specificity [211]. These same authors recently reported the significant increased expression of circulating miR-125b-1 and low levels of miR-203 in serum samples from untreated newly diagnosed children with ALL (*n* = 43), as detected by quantitative RT-PCR analysis [212]. They also showed higher levels of miR-125b-1 in T-ALL samples as compared to other ALL phenotypes [212]. More recently, circulating miR-652-3p was found downregulated in serum from ALL patients and levels reported as restored when patients attained in complete remission [47].

In AML patients, Fayyad-Kazan et al. [213] analyzed serum samples from a large cohort of newly diagnosed patients and compared them to normal samples from adult donors. After a two-phase selection and validation process, let-7d, miR-150, miR-339, and miR-342 were found downregulated, while let-7b and miR-523 were upregulated AML compared to control sera [213]. Other results have revealed the presence of two miRNAs, miR-150 and miR-342, significantly downregulated in the plasma of AML patients at diagnosis when compared to healthy controls [214]. Moreover, high serum miR-335 levels were associated with shorter RFS and OS. Furthermore, serum miR-335 and cytogenetic risk were identified as independent prognostic factors for both RFS and OS, suggesting miR-335 as a promising biomarker for pediatric AML [214,215]. Conversely, Zhao and colleagues [216] showed that miR-144 was markedly reduced in both the peripheral blood and bone marrow of AML patients. A similar pattern is commonly observed, miR-34a underexpression in AML patients with intermediate/poor risk cytogenetic and the M5 subtype [217]. More recently, low levels of miR-370 and miR-195 were described in sera of pediatric AML patients and associated with classification M7 subtype, unfavorable karyotype, and shorter RFS and OS [218,219].

In lymphoma, Lawrie et al. [207] showed miR-155, miR-210, and miR-21 in high levels in serum from DLBCL patients compared with healthy controls’ sera. Moreover, high miR-21 expression was associated with RFS [207], which was later confirmed by Chen et al. [220] in an independent cohort; thus far, it is the only circulating miRNA in DLBC that has shown consistent results and is now considered a biomarker for diagnosis [221].

More recently, the high levels of miR-155 and miR-22 in plasma from DLBCL patients were associated with shorter overall survival [222,223], while high levels of circulating miR-125b and miR-130a further demonstrated that they were involved in the recurrence, progression, and R-CHOP resistance [224]. Additionally, Khare et al. [199] described increased plasma levels of miR-124 and miR-532-5p, and decreased levels of miR-425, miR-141, miR-145, miR-197, miR-345, miR-424, miR-128, and miR-122 in plasma samples from patients with DLBCL through small-RNA sequencing.

For other lymphoma types, information is restricted to adult cases. MiR-221 has been described as elevated in plasma samples from T-LBL patients, with higher levels associated with a poorer long-term outcome [225]. Overexpression of miR-21 and miR-23a, on the other hand, has been associated with staging, WBC, upregulated serum lactate dehydrogenase (LDH) level, and tumor size ≥6 cm in BL, while miR-125b expression had an association with staging and upregulated serum LDH [226]. Additionally, a more recent study identified miR-25, miR-30a/d, miR-26b, miR-182, miR-186, miR-140*, and miR-125a to be upregulated, while miR-23a, miR-122, miR-93, and miR-144 were downregulated in HL [199].

### 4.2. Prognostic Use of MiRNAs

Despite substantial advancement in research and medicine, cancer remains a major public-health problem in our society. Thus, the utility of miRNA expression analysis as diagnostic and prognostic molecular markers is strongly supported. For example, analysis of 217 miRNAs from 334 samples including multiple human cancers provided expression signatures more accurate for cancer-subtype classification than expression-profiling of all known mRNAs does [227]. Additionally, as seen in previous sections, many miRNA dysregulations have also been associated with treatment response (Figure 2). Thereafter, many research groups have shown aberrant-expression profiles of miRNAs in a broad variety of human malignant cancers, especially hematological cancer (Tables S1 and S2). Furthermore, miRNA analysis has some benefits because these molecules are highly resistant to degradation and their expression levels can be obtained in a few hours with small biological samples [9].

### 4.3. Therapeutic Use of MiRNAs

The progress of miRNAs analysis as molecular markers creates hope for personalized cancer treatments by miRNA modulation. Unfortunately, miRNAs are still not druggable, and clinics are far from reality due to a variety of challenges. Nonetheless, local delivery through encapsulation in lipidic or polymer nanoparticles, or ultrasound-mediated microbubble formulations and hyaluronic acid (HA)/protamine sulfate (PS) interpolyelectrolyte complexes, has shown promising results in mice models [228,229,230,231,232,233]. Viral vectors for delivering miRNAs into cells have also been widely used in preclinical studies; nonetheless, their safety remains controversial mainly because of lack of safety (i.e., lentivirus), off-target effects, or immune responses [234].

So far, only two strategies have entered clinical trials, though none of them involved hematologic cancer. Miravirsen (Satnaris Pharma^®^), a locked nucleic acid-modified DNA antisense oligonucleotide that targets the liver-specific miR-122 has demonstrated antiviral activity against hepatitis C and, at phase 2, no dose-limiting adverse events [233,235]. More recently, Mirna Therapeutics Inc. began treating patients with advanced solid tumors with MRX34^®^, a liposomal injection carrying encapsulated miR-34 showing acceptable safety and evidence of antitumor activity in a subset of patients, despite some liposome-related toxicities [236].

## 6. Concluding Remarks

Targeted therapies have distinctly transformed the treatment of cancer over the past decade. The utility of miRNA-expression analysis as diagnostic and prognostic molecular markers is strongly supported and, in the near future, it may impact the treatment of hematological cancer.

## Supplementary Materials

Supplementary materials can be found at http://www.mdpi.com/1422-0067/19/9/2688/s1.

## Abbreviations

ABC	Activated B-cell-like
ALCL	Anaplastic Large-Cell Lymphoma
ALL	Acute Lymphoblastic Leukemia
AML	Acute Myeloid Leukemia
BL	Burkitt Lymphoma
B-LBL	B-cell Lymphoblastic Lymphoma
cALL	Childhood ALL
CML	Chronic Myeloid Leukemia
CNS	Central nervous System
CYT	Cytarabin
DAUNO	Daunorrubicin
DEXA	Dexamethasone
DFS	Disease-Free Survival
DLBCL	Diffuse Large B-cell lymphoma
eBL	Endemic Burkitt Lymphoma
EBNA1	Epstein–Barr nuclear antigen 1
EBV	Epstein–Barr virus
EFS	Event-Free Survival
GCB	Germinal center B cell-like
HL	Hodgkin Lymphoma
INZ	Inauhzin
JMML	Juvenile Myelomonocytic Leukemia
l-ASP	l-asparaginase
LDH	Lactate dehydrogenase
LL	Lymphoblastic Lymphoma
miR	Micro RNA
MiRNAs	Micro RNAs
MTX	Methotrexate
NHL	Non-Hodgkin Lymphoma
OS	Overall Survival
PMLBCL	Primary Mediastinal Large B Cell Lymphoma
PRED	Prednisone
PREDS	Prednisolone
RFS	Relapse-Free Survival
RT-PCR	Reverse Transcription Polymerase Chain Reaction
T-LBL	T-cell Lymphoblastic Lymphoma
VIN	Vincristine
WHO	World Health Organization
