# Supplementary material for: MiRNA Dysregulation in Childhood Hematological Cancer
Source: Int J Mol Sci. 2018 Sep 10;19(9):2688. doi: 10.3390/ijms19092688 (PMC6165337; doi:10.3390/ijms19092688)
Supplement: Supplementary file 1 [file ijms-19-02688-s001.zip › supplementary tables/Supplemental table 1.docx]

| **Supplemental table 1:** Compilation of dysregulated miRNAs in pediatric leukemias | | |  |
| --- | --- | --- | --- |
|  |  |  |  |
| **miRNA** | **Expression in tumor** | **Function** | **References** |
|  |  |  |  |
| **Acute Lymphoblastic Leukemia** | |  |  |
| let-7b | down | Associated with the MLL-rearranged subtype | [16, 18] |
| miR-let-7e | down | No clinical associations described / not tested functionally | [18] |
| miR-7 | up | CNS relapse | [23, 40] |
| miR-17-3p | down | Decreased in T-cell Subtype | [18] |
| miR-17-5p | down | Decreased in T-cell Subtype | [18] |
| miR-18 | down | No clinical associations described / not tested functionally | [18-19,21,24] |
| miR-18a | up | Predicts prednisone response | [23] |
| miR-21 | up | No clinical associations described / not tested functionally | [19–23] |
| miR-23a | Up / down | Prognostic value | [54] |
| miR-24 | down | Associated with PAR1-deleted subtype | [18,20,29] |
| miR-27a | Up / down | Prognostic value | [54,59] |
| miR-29c-3p | down | Decreased in T-cell Subtype | [18,20,28-29] |
| miR-30e-5p | up | No clinical associations described / not tested functionally | [18] |
| miR-31 | down | Associated with PAX5-deleted subtype | [18,20,29] |
| miR-34 | up | OncomiR / treatment resistance | [19-23,37-38] |
| miR-34b | up | No clinical associations described / not tested functionally | [18] |
| miR-92a-3p | down | Decreased in T-cell subtype | [18] |
| miR-99a | up / down | Drug resistance | [18,20-21,24-25,32,34,45-46] |
| miR-100 | down | Associated with ETV6/RUNX1 translocation | [18-19,21,24,28-29] |
|  | up / down | Vincristine and daunorrubicin resistance | [27,45,46] |
|  | up | Circulating biomarker | [215] |
| miR-124 | up | Prednisone and glucocorticoid resistance | [41] |
| miR-125b | down | Tumor suppressor / treatment resistance | [18,27,45,46] |
|  | up | Associated with ETV6/RUNX1 translocation | [18,29-30] |
| miR-125-b1 | up | Circulating biomarker | [216] |
| miR-126 | down | Vincristine and daunorrubicin resistance / CNS relapse | [27] |
| miR-128 | up | No clinical associations described / not tested functionally | [18,20-23,27,31] |
|  | down | Associated with PAX5-deleted subtype | [18,29] |
| miR-128a | up | Associated with the MLL-rearranged subtype | [16,18] |
| miR-128b | up | Treatment resistance / prognostic value | [16,39, 43,54] |
| miR-142 | up | No clinical associations described / not tested functionally | [18–23] |
| miR-142-3p | up | No clinical associations described / not tested functionally | [17] |
| miR-145 | down | No clinical associations described / not tested functionally | [19,21,24,25] |
| miR-146a | up | Circulating / diagnosis | [19–23,215] |
| miR-150 | up | No clinical associations described / not tested functionally | [18] |
| miR-151 | down | Worse RFS | [61] |
| miR-181 | up | OncomiR / prognostic value | [18-23,54] |
| miR-181a | down | Tumor suppressor / survival | [18-19,21,24] |
| miR-181b | up | Associated with the MLL-rearranged subtype | [18,20,27,31] |
| miR-193 | up | No clinical associations described / not tested functionally | [18] |
| miR-193a | up | Predicts prednisone response | [23] |
| miR-195 | up | No clinical associations described / not tested functionally | [19–23] |
| miR-196 | up | Associated with ETV6/RUNX1 translocation | [18,29-30] |
| miR-196a | up | Circulating / diagnosis | [215] |
| miR-196b | down | No clinical associations described / not tested functionally | [18-19,21,24,25] |
|  | up | Associated with T-cell Subtype | [19,32–34] |
| miR-198 | up | CNS relapse | [40] |
| miR-203 | down | Circulating / diagnosis | [216] |
| miR-210 | up | Good prognosis / treatment response | [53,60] |
| miR-214-3p | down | Decreased in T-cell Subtype | [18,28] |
| miR-214-5p | down | Decreased in T-cell Subtype | [18,28] |
| miR-218 | up | Predicts prednisone response | [23] |
| miR-221 | down | Glucocorticoids resistance | [43] |
| miR-222 | up / down | OncomiR / CNS relapse | [17,40] |
| miR-223 | up | Treatment resistance / prognostic value | [39,54] |
|  |  | Associated with ETV6/RUNX1 translocation | [18,29-30] |
| miR-335 | down | Predicts prednisone response | [43] |
| miR-339 | up | No clinical associations described / not tested functionally | [17] |
| miR-345 | down | CNS relapse | [40] |
| miR-365 | up | No clinical associations described / not tested functionally | [18] |
| miR-373 | down | No clinical associations described / not tested functionally | [17-18] |
| miR-451 | down | Worse RFS | [17,61] |
| miR-532 | up | Predicts prednisone response | [23] |
| miR-542 | up | Associated with PAR1-deleted subtype | [18,28] |
| miR-550 | up | Predicts prednisone response | [23] |
| miR-551a | down | CNS relapse | [40] |
| miR-582 | up | No clinical associations described / not tested functionally | [18] |
| miR-595 | polymorphism | Methotrexate response | [50–52] |
| miR-625 | up | Predicts treatment response | [23,27] |
| miR-633 | up | Predicts prednisone response / CNS relapse | [23,40] |
| miR-638 | up | Predicts prednisone response | [23] |
| miR-652-3p | down | Resistance to vincristine and cytarabin / circulating | [47] |
| miR-708 | up | OncomiR / survival | [18-23,54] |
|  | up | Associated with ETV6/RUNX1 translocation | [18,29-30] |
|  | down | Decreased in T-cell and PAX5-deleted Subtypes | [20] |
| miR-1206 | polymorphism | Methotrexate response | [50–52] |
| miR-1290 | up | Worse RFS | [61] |
| miR-3117-3p | polymorphism | Vincristine-induced neurotoxicity | [49] |
| miR-5189 | polymorphism | Methotrexate response | [50–52] |
| miR-6083 | polymorphism | Methotrexate response | [50–52] |
|  |  |  |  |
| **Acute Myeloid Leukemia** | |  |  |
| let-7b | up | Circulating biomarker | [217] |
| let-7d | down | Circulating biomarker | [217] |
| miR-9 | down | Associated with t(8;21) subtype | [65] |
| miR-29a | down | Associated with MLL rearranged cases / Poor prognosis indicator | [74,76] |
| miR-34a | down | Circulating biomarker / associated with M5 subtype | [221] |
| miR-34b | down | No clinical associations described / not tested functionally | [79] |
| miR-99a | up | No clinical associations described / not tested functionally | [63] |
| miR-100 | up | Associated with MLL / poor RFS | [73,78] |
| miR-106~25 | up | Associated with the MLL-rearranged subtype | [80] |
| miR-122 | down | No clinical associations described / not tested functionally | [78] |
| miR-125 | up | Associated with the MLL-rearranged subtype | [77] |
| miR-126 | down | Tumor supressor / associated with MLL-rearranged subtype | [65,78,82,83] |
| miR-144 | down | Circulating biomarker | [220] |
| miR-146a | down | Associated with MLL-rearranged subtype | [78] |
| miR-150 | down | Circulating biomarker | [217-218] |
| miR-155 | up | Diganostic biomarker / associated with FLT3 and NPM1-mutated cases / adverse prognosis in NK-AML | [67,68,69,76] |
| miR-181a/b | down | Associated with MLL-rearranged subtype / G1/S transition | [65,78,81] |
| miR-187 | up | Associated with MLL-rearranged subtype | [65] |
| miR-192-194 | down | Associated with inv(16) subtype | [65] |
| miR-195 | down | Circulating biomarker | [222-223] |
| miR-196a | up | Associated with M4-M5, MLL, NPM1 or FLT3 / ITD subgroups | [67,68,76] |
|  | down | Associated with CEBPA-mutated cases | [76] |
| miR-196b | up | Associated with M4-M5, MLL, NPM1 or FLT3 / ITD subgroups | [67,68,76] |
|  | down | Associated with CEBPA-mutated cases | [76] |
| miR-335 | up | Circulating biomarker / shorter RFS and OS | [218-219] |
| miR-339 | down | Circulating biomarker | [217] |
| miR-342 | down | Circulating biomarker | [217-218] |
| miR-370 | down | Circulating biomarker | [222-223] |
| miR-375 | up | Poor RFS | [73] |
| miR-500a | down | Associated with inv(16) subtype | [65] |
| miR-523 | up | Circulating biomarker | [217] |
| miR-582 | down | Associated with t(8;21) Subtype | [65] |
| miR-663 | down | No clinical associations described / not tested functionally | [64] |
| miR-1331 | down | Associated with MLL-rearranged subtype | [65] |
|  |  |  |  |
| **Chronic Myeloid Leukemia** | |  |  |
| miR-10 | down | No clinical associations | [90] |
| miR-99a | up | No clinical associations described / not tested functionally | [89] |
| miR-146b | down | No clinical associations | [90] |
| miR-203 | down | Regulates ABL1 and BCR / ABL1 expression | [87,88] |
|  |  |  |  |
| **Juvenile Myelomonocytic Leukemia** | |  |  |
| let-7 | down | Associated with fetal-like subgroup with LIN28 overexpression | [95] |
| let-7a-5p | down | Associated with PTPN11, KRAS and NRAS subtypes | [100] |
| let-7d-5p | down | Associated with PTPN11, KRAS and NRAS subtypes | [100] |
| let-7f-5p | down | Associated with PTPN11, KRAS and NRAS subtypes | [100] |
| let-7g-5p | down | Associated with PTPN11, KRAS and NRAS subtypes | [100] |
| miR-15a | up | Associated with PTPN11 mutated subtype | [98] |
| miR-23a-3p | up | Associated with PTPN11, KRAS and NRAS subtypes | [100] |
| miR-26a-5p | down | Associated with PTPN11, KRAS and NRAS subtypes | [100] |
| miR-29a-3p | down | Associated with PTPN11, KRAS and NRAS subtypes | [100] |
| miR-29b-3p | down | Associated with PTPN11, KRAS and NRAS subtypes | [100] |
| miR-30b-5p | down | Associated with PTPN11, KRAS and NRAS subtypes | [100] |
| miR-34b | up | Not confirmed by others | [97] |
| miR-146b-5p | down | Associated with PTPN11, KRAS and NRAS subtypes | [100] |
| miR-148a-3p | down | Associated with PTPN11, KRAS and NRAS subtypes | [100] |
| miR-222-3p | up | Associated with PTPN11, KRAS and NRAS subtypes | [100] |
| miR-223 | up | Associated with PTPN11 mutations | [98] |
| miR-224-5p | up | Associated with PTPN11, KRAS and NRAS subtypes | [100] |
| miR-320e | up | Associated with PTPN11, KRAS and NRAS subtypes | [100] |
| miR-338-3p | up | Associated with PTPN11, KRAS and NRAS subtypes | [100] |
| miR-342-3p | down | Associated with PTPN11, KRAS and NRAS subtypes | [100] |
| miR-486 | up | Regulator of PTEN and FOXO1 | [96] |
| miR-494 | up | Associated with PTPN11, KRAS and NRAS subtypes | [100] |
| mir-548ai | up | Associated with PTPN11, KRAS and NRAS subtypes | [100] |
| miR-575 | up | Associated with PTPN11, KRAS and NRAS subtypes | [100] |
| miR-630 | up | Associated with PTPN11, KRAS and NRAS subtypes | [100] |
| miR-3195 | up | Associated with PTPN11, KRAS and NRAS subtypes | [100] |
| miR-4508 | up | Associated with PTPN11, KRAS and NRAS subtypes | [100] |
| miR-150-5p | down | Associated with PTPN11, KRAS and NRAS subtypes | [100] |
| miR-1260a | down | Associated with PTPN11, KRAS and NRAS subtypes | [100] |
| miR-4454 | down | Associated with PTPN11, KRAS and NRAS subtypes | [100] |

Up – up-regulated; down- down-regulated; MLL – Lysine Methyltransferase; CNS – central nervous system; RFS – relapse-free survival; PAR1 – Phy Rapidly Regulated 1; PAX5 –Paired Box 5 ; ETV6 – ETS variant 6; RUNX1 – Runt Related Transcription Factor 1; M5 subtype – acute monocytic leukemia; NK-AML – natural killer cells in acute myeloid leukemia; inv (16) inversion of chromosome 16; M4 subtype – acute myelomonocytic leukemia; NPM1 – Nucleophosmin 1; FLT3 – FMS liked tyrosin 1; ITD- internal tandem duplication; OS – overall survival; t(8;21) – translocation between chromosomes 8 and 21; CEBPA - CCAAT enhancer binding protein alpha; ABL1 – ABL proto-oncogene 1; BCR – BCR, RhoGEF and GTPase activating protein; PTPN11 – Protein Tyrosine Phosphatase, non-receptor type 11; KRAS- KRAS proto-oncogene; NRAS – NRAS proto-oncogene; PTEN – Phosphatase and Tensin homolog; FOXO1 – forked head box O1.
