# Supplementary material for: MiRNA Dysregulation in Childhood Hematological Cancer
Source: Int J Mol Sci. 2018 Sep 10;19(9):2688. doi: 10.3390/ijms19092688 (PMC6165337; doi:10.3390/ijms19092688)
Supplement: Supplementary file 1 [file ijms-19-02688-s001.zip › supplementary tables/Supplemental table 2.docx]

| **Supplemental table 2**: Compilation of dysregulated miRNAs in pediatric lymphomas | | |  |
| --- | --- | --- | --- |
|  |  |  |  |
| **miRNA** | **Expression in tumor** | **Function** | **References** |
|  |  |  |  |
| **Burkitt Lymphoma** | |  |  |
| let-7 family | down | Associated with eBL subtype | [143] |
| miR-BART | expression | OncomiR / targets interleukin-6 receptor (IL-6R) | [123-124] |
| miR-10a-5p | down | eBL localized in the jaw | [144] |
| miR-17 | up | Predictor of shortened OS | [135] |
| miR-17~92 | up | Associated with t(8;14) / associated with eBL | [106,130-131,143] |
| miR-18b | up | Associated with t(8;14) | [106] |
| miR-20b | up | Associated with t(8;14) | [106] |
| miR-23a | down | Associated with MYC translocation | [128] |
| miR-26a | down | Tumor suppressor | [128,136,137] |
| miR-28 | down | Tumor suppressor | [136,137] |
| miR-29a | up | Tumor suppressor MYC translocation-positive cases / targets DNMT1 | [134] |
| miR-29b | up | Tumor suppressor MYC translocation-positive cases / targets DNMT2 | [128,134] |
| miR-30d | down | Reppressed by MYC | [128] |
| miR-106a | up | Associated with MYC translocation | [106] |
| mir-142 | up | Targets interleukin-6 receptor (IL-6R) | [125-126] |
| miR-146a | down | Reppressed by MYC | [128] |
| miR-146b-5p | down | Reppressed by MYC | [128] |
| miR-150 | down | Tumor suppressor / targets MYB and survivin | [138-139] |
| miR-155 | up | Observed in EBV positive cases | [113-115] |
|  | down | Targets SHIP1, Nuclear Interactor of ARF, MMD2 and VEGF-A in ARPE-19 cells | [111-113,116] |
| miR-181b | down | Tumor suppressor / targets FAMLF | [140-141] |
| miR-197 | up | Targets interleukin-6 receptor (IL-6R) | [125-126] |
| miR-221 | down | No clinical associations described / not tested functionally | [128] |
| miR-513a | down | Associated with MYC negative cases | [134] |
| miR-520a | down | Tumor supressor / targets AKT1 and NFkB | [129] |
| miR-628-3p | down | Associated with MYC negative cases | [134] |
|  |  |  |  |
| **Diffuse Large B- cell lymphoma** | |  |  |
| miR-17~92 | up | Associated with GCB subtype | [156] |
| miR-21 | up | Associated with ABC subtype / circulating biomarker / associated with RFS | [226-227] |
| miR-22 | up | Circulating biomarker | [149,150,211,224-225] |
| miR-28 | up | Better outcome | [155] |
| miR-34a | down | Targets FOXP1 | [159] |
| miR-106a | up | Associated with GCB subtype | [157] |
| miR-125a/b | up | Target TNFαIP3 / poor prognosis / treatment response | [158,228] |
| miR-146a | up | Associated with ABC subtype / better prognosis / treatment response | [149,150,154] |
| miR-146b | up | Associated with ABC subtype | [149,150] |
| miR-155 | up | Associated with ABC subtype / better prognosis / treatment response / circulating biomarker | [149,150-151,154,211, 226-227] |
| miR-181b | up | Associated with GCB subtype | [157] |
| miR-210 | up | Circulating biomarker | [211] |
| miR-214 | up | Better outcome | [155] |
| miR-221 | up | Associated with ABC subtype | [149,150] |
| miR-222 | up | Associated with ABC subtype / worse prognosis | [149,150,153] |
| miR-339* | up | Better outcome | [155] |
| miR-500 | up | Associated with ABC subtype | [149,150] |
| miR-574 | up | Associated with ABC subtype | [149,150] |
| miR-574* | up | Associated with ABC subtype | [149,150] |
| miR-5586 | up | Better outcome | [155] |
|  |  |  |  |
| **Anaplastic large cell lymphoma** | |  |  |
| miR-16 | down | Tumor suppressor / targets VEGF | [179] |
| miR-17~92 | up | OncomiR / associated with ALK+ subtype | [180-181] |
| miR-20b | up | No clinical associations described / not tested functionally | [180] |
| miR-22 | down | No clinical associations described / not tested functionally | [180] |
| miR-29a-b | down | Tumor suppressor / associated with ALK+ subtype | [183] |
| mir-101 | down | Targets mTOR / tumor suppressor in ALK+ subtype | [180] |
| miR-125b | down | No clinical associations described / not tested functionally | [180] |
| miR-135b | up | Associated with ALK+ subtype | [177,178] |
| miR-146a | down | Associated with ALK+ subtype | [177,178] |
| miR-150 | down | No clinical associations described / not tested functionally | [180] |
| miR-155 | down | Associated with ALK+ subtype | [177,178] |
| miR-512* | up | Associated with ALK+ subtype | [177,178] |
| miR-708 | up | Associated with ALK+ subtype | [177,178] |
| miR-886 | up | Associated with ALK+ subtype / targets BAX | [177,178] |
| miR-886* | up | Associated with ALK+ subtype | [177,178] |
|  |  |  |  |
| **T-cell lymphoblastic lymphoma** | |  |  |
| miR-17 | up | Unfavorable prognosis | [192] |
| miR-19 | up | Unfavorable prognosis | [192] |
| miR-21 | up | Circulating biomarker / prognostic value | [231] |
| miR-23a | up | Circulating biomarker / prognostic value | [231] |
| mir-125b | up | Circulating biomarker / prognostic value | [231] |
| miR-200a-3p | down | Targets TP53 | [195] |
| miR-203a | down | Targets E2F1 | [195] |
| miR-205-5p | down | Targets E2F1 | [195] |
| miR-221 | up | Circulating biomarker / poor outcome | [230] |
| miR-221-3p | up | Targets CDKND1 | [195] |
| miR-222-3p | up | Targets CDKND2 | [195] |
| miR-241 | up | No clinical associations described / not tested functionally | [193] |
| miR-374b | down | Tumor suppressor / targets AKT1 and WNT16 | [194] |
| miR-375 | down | Targets TP53 | [195] |

Up – up-regulated; down- down-regulated; eBL – endemic Burkitt Lymphoma; OS – overall survival; t(8;14) - translocation between chromosomes 8 and 14; DNMT1 - DNA methyltransferase 1; DNMT2 - DNA methyltransferase 2; MYC – MYC proto-oncogene; MYB – MYB proto-oncogene; EBV - Epstein–Barr virus; SHIP1 – Inositol polyphosphate-5-phosphatase D; ARF – known as cyclin dependent kinase inhibitor 2A; MMD2 – Monocyte to macrophage differentiation associated 2; VEGF-A- Vascular endothelial growth factor; AKT1 – AKT serine/threonine kinase 1; GCB subtype – germinal center B-cell subtype; ABC subtype – activated B-cell subtype; RFS – relapse-free survival; FOXP1 – Forked head box P1; ALK – ALK receptor tyrosine kinase; mTOR – mechanistic target of rapamycin; BAX – BCL2 associated X; TP53 – tumor protein p53; E2F1 – E2F transcription factor 1; CDKND – cyclin dependent kinase inhibitor; WNT16 – wnt family member 16.
